# GH Resistance Is a Component of Idiopathic Short Stature: Implications for rhGH Therapy

**DOI:** 10.3389/fendo.2021.781044

**Published:** 2021-12-10

**Authors:** Martin O. Savage, Helen L. Storr

**Affiliations:** Centre for Endocrinology, William Harvey Research Institute, Barts and the London School of Medicine & Dentistry, Queen Mary University of London, London, United Kingdom

**Keywords:** growth, short stature, growth hormone resistance, genetic defects, idiopathic short stature, growth hormone therapy

## Abstract

Idiopathic short stature (ISS) is a term used to describe a selection of short children for whom no precise aetiology has been identified. Molecular investigations have made notable discoveries in children with ISS, thus removing them from this category. However, many, if not the majority of children referred with short stature, are designated ISS. Our interest in defects of GH action, i.e. GH resistance, has led to a study of children with mild GH resistance, who we believe can be mis-categorised as ISS leading to potential inappropriate management. Approval of ISS by the FDA for hGH therapy has resulted in many short children receiving this treatment. The results are extremely variable. It is therefore important to correctly assess and investigate all ISS subjects in order to identify those with mild but unequivocal GH resistance, as in cases of PAPP-A2 deficiency. The correct identification of GH resistance defects will direct therapy towards rhIGF-I rather than rhGH. This example illustrates the importance of recognition of GH resistance among the very large number patients referred with short stature who are labelled as ‘ISS’.

## Introduction

The term idiopathic short stature (ISS) was first applied to short children without a known aetiology over 35 years ago, long before the era of molecular investigation. A current definition of ISS will be discussed below. However, ISS is not a definitive diagnosis. Its original use as a description of children with short stature, who did not have GH deficiency, served a purpose in its day, but now clinicians take the investigation of such children further, with new opportunities presenting a realistic chance of identifying causative pathogeneses (1). As precision medicine attempts to personalise diagnosis and therapy, new genetic discoveries in the GH-IGF-I axis and growth plate chondrogenesis provide opportunities for more precise diagnosis (1).

## GH Resistance

GH resistance in children embraces a range of defects in the GH-IGF-I axis characterised by an abnormality in the action of GH (2). In the context of child with short stature, it is the milder forms of GH resistance, which tend to be confused with ISS. The extreme or ‘classical’ form of GH resistance, so-called Laron syndrome, is relatively easy to diagnose because of its extreme phenotype and is unlikely to be confused with ISS. However milder or ‘non-classical’ GH resistance disorders might overlap clinically and thus be mis-categorised as ISS. In 2019, an extensive review of mild or ‘non-classical’ abnormalities of GH action was published by our group (3). These findings will be summarised below together with a hypothesis that in many cases mild GH resistance disorders may be mis-diagnosed as ISS.

## The Origin of the ISS Designation

The diagnosis of GH deficiency in children entered clinical practice in the late 1960s with the demonstration of GH release following stimulation by insulin-induced hypoglycaemia or acute administration of glucagon and other GH secretagogues (4). GH stimulation tests permitted diagnosis of GH deficiency and thereby separated GH deficient patients from those with similar appearance but normal GH secretion. Towards the end of the era of administration of pituitary-derived hGH, which terminated in 1985 due to the Creutzfeldt-Jakob epidemic, the anticipation of the availability of recombinant hGH (rhGH), first synthesized in 1979 (5), led to short and long-term studies of hGH therapy in subjects with so-called ‘normal variant short stature’ (6) or labelled as ‘short normal’ children (7). A conference, convened at the NIH in November 1983 to discuss the future use of rhGH in short children without GH deficiency, concluded that in a society that ‘values tallness’, controlled research studies of rhGH in such patients were authorized (8). At that meeting, ISS as a diagnostic group acquired scientific respectability.

## Current Definition of ISS and Its Sub-Classification

The definition of ISS is clinically important because inclusion of a child with short stature within this designation may, in certain societies where ISS is approved for hGH therapy, provide an indication for this treatment. ISS is currently defined as short stature with height <-2 SDS, normal birth size (birth weight and length >-2 SDS), absence of abnormal physical features and normal general screening investigations, normal body proportions and absence of major dysmorphic features (9). It should be noted that the above definition of ISS is different from the height criterion of <-2.25 SD approved by the FDA for rhGH therapy (10).

The components of ISS were critically appraised in two reviews by Wit et al. in 2008 (10, 11). A Consensus Statement on ISS management was also published in 2008 (12). ISS was subdivided into familial short stature (FSS) with normal or delayed bone age and non-familial short stature (NFSS) with normal or delayed bone age (9–11). The definitions proposed for FSS and NFSS are based on the calculation of ‘conditional’ target height (cTH), which is adjusted for the correlation between maternal and paternal heights, so-called assortative mating, and for the correlation between children’s height SDS and mid-parental height SDS (13). The definition of FSS is Height SDS = cTH SDS ± 1.6 and of NFSS, Height SDS < cTH SDS -1.6 based on the fact that 95% of healthy children have Height SDS = cTH SDS ± 1.6 (the TH range). It should be noted that FSS may co-exist with constitutional delay of growth and puberty (CDGP) in the same patient, who might present earlier with short stature. Most children with CDGP seen in a growth disorders clinic have at least one parent who is short.

It is likely that in most subjects with FSS, the short stature is related to the inheritance of polygenic variants from both parents with multiple small negative effects on height. However, a copy number variant (CNV) or monogenic defect is also possible, particularly if there is a pattern of dominant inheritance, notably from one parent. The inheritance of multiple variants in the same or different growth-related pathways may occur (14). In children with NFSS and a slow tempo of growth constitutional delay of growth and puberty is statistically the most likely diagnosis, particularly if bone age is delayed and the family history is positive for delayed puberty. However, also a recessive or *de novo* pathogenic gene variant or CNV should be considered. It is in the NFSS group that defects associated with adult height below parental target height are most likely to occur.

**Figure 1:**
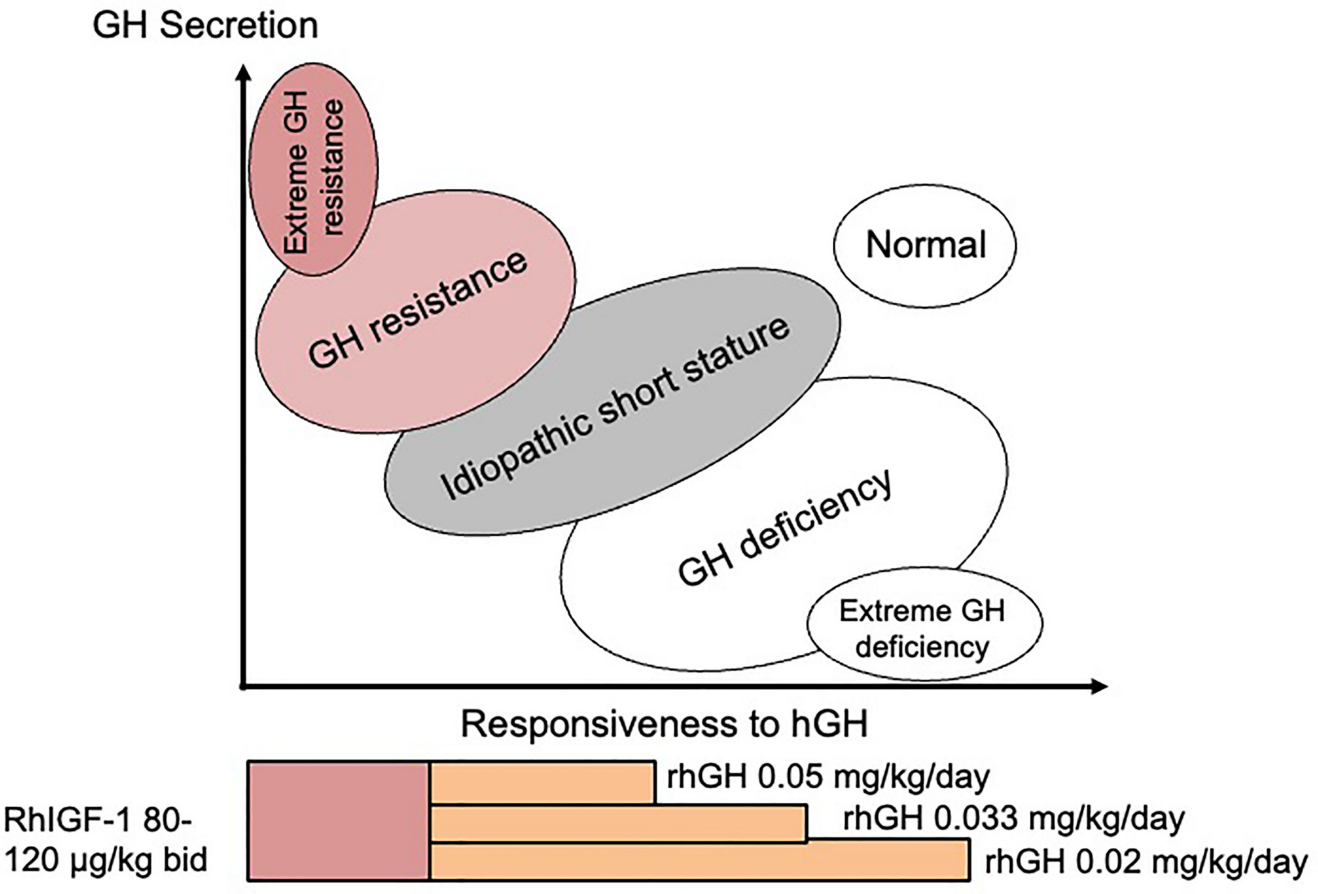
The continuum model showing the relationship between disorders of the GH-IGF-I axis and responsiveness to hGH therapy. Suggested therapy with doses of rhGH and rhIGF-I relating to the different disorders is also shown.

At the time when the GH-IGF-I axis was considered to be the major influence for growth regulation, ‘ISS’ was used to describe children who fell between GH deficiency and GH insensitivity in the so-called GH-IGF-I axis continuum model (**Figure 1**) (15). According to this model, ISS subjects should have a normal physiological equilibrium between GH sensitivity and GH deficiency, which is the case for those with FSS, where no endocrine defect in the child or parents has been identified. However, since the discovery that most genes associated with normal linear growth have no direct relationship with the GH-IGF-I axis (16), it appears more useful to think in terms of another conceptual framework for understanding short and tall stature that is centred not on the GH-IGF-I axis, but rather on the growth plate (17). In the 21st Century, 35 years after its inception, ISS therefore describes a highly heterogeneous group of short patients and should no longer be used as a single definitive diagnostic category (1).

## Assessment of Patients With Short Stature and Investigation of ISS and GH Resistance

**Figure 2:**
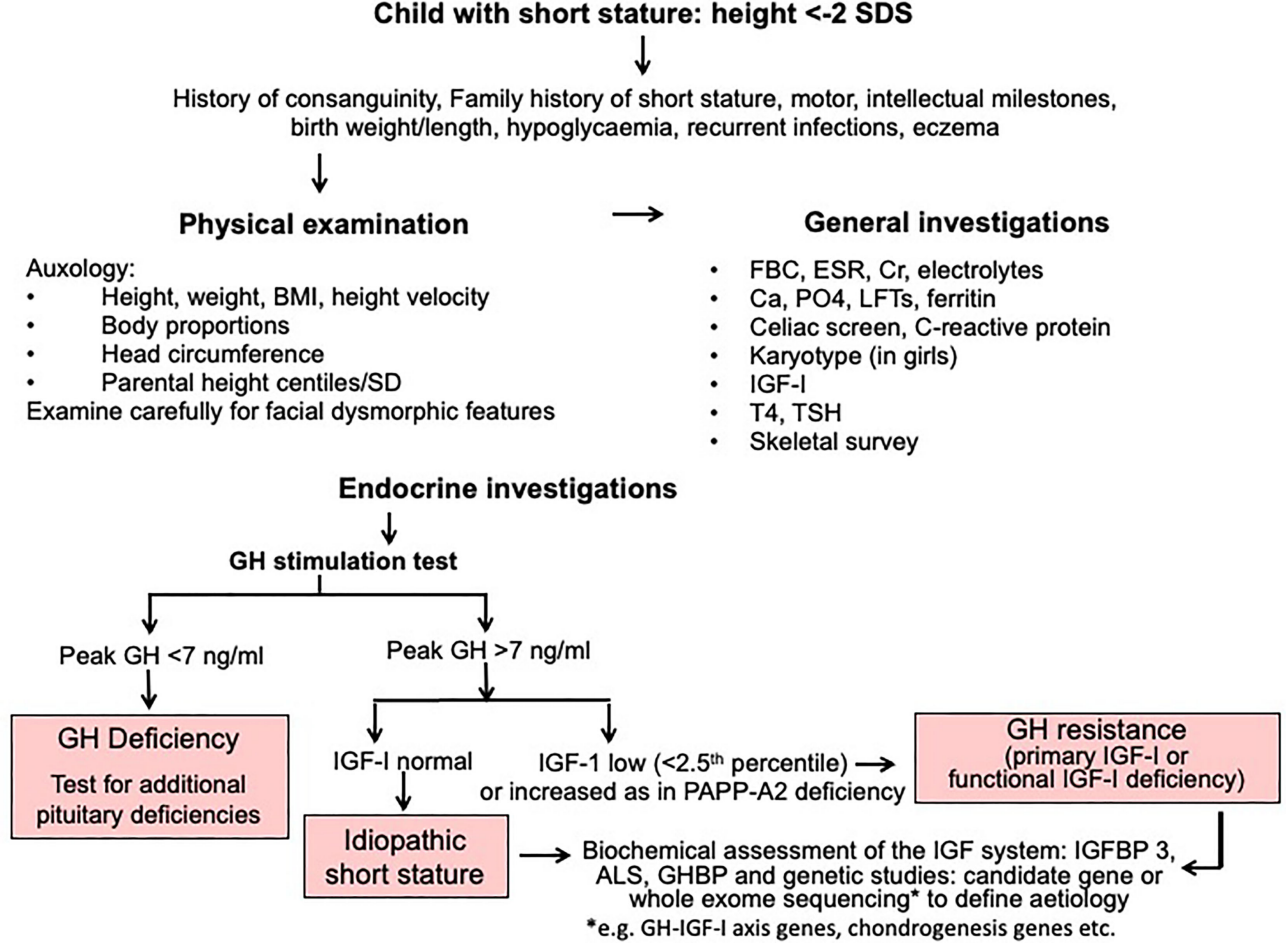
Algorithm for investigation of short stature, idiopathic short stature and GH resistance.

A diagnostic algorithm for investigation of short stature is shown in **Figure 2**. The three key approaches of clinical assessment, endocrine evaluation and genetic analysis should have equal status in the hierarchy of assessment variables. We resist the suggestion to give genetic analysis more prominence (14) at the expense of clinical assessment, because clinical skills are crucial in terms of identifying a phenotype and taking a valuable history (1). The classification of growth disorders into primary growth plate defects and secondary abnormalities affecting growth plate function has redressed the balance of probability of correct pathogenesis away from the GH-IGF-I axis towards defects of chondrogenesis (1, 17). A diagnosis of GH resistance can be made following evaluation of GH secretion and the IGF system (**Figure 2**), however the precise molecular pathogenesis will require next generation sequencing using either candidate gene or whole exome sequencing techniques (1, 2, 14).

## Endocrine Abnormalities in Patients Initially Considered to Have ISS

In the 1980s and 1990s the study of childhood linear growth focused on the function of different components of the GH-IGF-I axis and enormous progress in the understanding of this axis was made (18). The original somatomedin hypothesis, published in 1957 (19), was up-dated 50 years later (20) showing that the IGF system played a key role in growth regulation with both circulating and peripherally produced IGF-I having individual roles (21). IGF-I deficiency was reported to occur in a proportion of short patients with normal GH secretion (22), which placed some ISS patients in an intermediate position between GH deficiency and GH resistance, although some overlap existed.

## GH Resistance as a Component of ISS

Evidence has accumulated that some ISS patients have a degree of functional GH resistance (22) with a broad range of generation of IGF-I in response to GH. The important study by Cohen et al. in 2007 reported that in some ISS patients high doses of rhGH were needed to reach a serum IGF-I concentration of +2 SD (23). Evidence of subnormal generation of IGF-I was also demonstrated in the elegant studies by Buckway and Selva of responses in the IGF-I generation test (IGFGT). Compared to normal control subjects, ISS patients had basal IGF-I levels in the lower half of the normal range and after GH stimulation on days 5 and 8 of the IGFGT, IGF-I levels were significantly lower than normal, regardless of GH dose (24, 25). It should be noted that the IGFGT has suffered from a lack of standardization, both of dose of rhGH used and duration of stimulation (10). Similarly, normative data have not been established and the routine use of the IGFGT is not recommended in the investigation of ISS (12). The hormonal findings of possible IGF-I deficiency in ISS challenge the definition that states that ISS is associated with no endocrine abnormality. The molecular basis of these findings was not apparent at that time.

## Variants in Genes Regulating GH Action With Phenotypes Consistent With ISS

ISS patients may have variable GH resistance and IGF-I concentrations (10) and consistent with this, a proportion have a diminished response to rhGH therapy (11, 15). Therefore, it has been suggested that less deleterious *GHR* gene defects may cause ISS associated with features of GH resistance (26). Numerous studies of ISS cohorts have reported heterozygous *GHR* variants occurring with a frequency ranging from 5 to 15.5% (27). It has also been noted that *GHR* sequence changes are common in children with ISS with many also identified in control subjects and normal stature family members (3).

Since the inception of genetic investigations of short stature phenotypes in the late 1980s, a number of pathogenic variants have been discovered in children labelled as having ISS. Mild forms of GH resistance can be broadly divided into three categories; 1) aberrations of GH signalling caused by homozygous or heterozygous variants of genes encoding the GH receptor (*GHR*) or Signal Transducer And Activator of Transcription 5B (*STAT5B*) (3, 28); 2) defects of IGF-I secretion (*IGFI*), transport (*IGFALS*) and bioavailability (*PAPPA2*) (3, 29, 30) and; 3) IGF-I insensitivity (*IGFIR*) (31). A summary of phenotypic and endocrine features of genetic defects consistent with ISS is shown in **Table 1**.

**Table 1:** Summary of phenotypic and biochemical features of defects causing GH resistance originally labelled as ISS.

Phenotype	Gene defect						
	*GHR heterozygous dominant negative*	*GHR pseudo-exon*	*STAT5B heterozygous dominant negative*	*IGFI*	*IGF2 (heterozygous variants)*	*IGFALS*	*PAPPA2*
Short stature	**+**	**+**	**+**	**+**	**+**	**+**	**+**
Mid-face hypoplasia	**–**	*	**+/–**	**–**	**–**	**–**	**–**
Other facial dysmorphism	**–**	**–**	**–**	**+** **Micrognathia**	**+**	**–**	**+** **Long thin nose** **Small chin**
Deafness	**–**	**–**	**–**	**+/–**	**–**	**–**	**–**
Microcephaly	**–**	**–**	**–**	**+**	**–**	**–**	**+/–**
Intellectual deficits	**–**	**–**	**–**	**+**	**–**	**–**	**–**
Pubertal delay	**–**	**–**	**+/–**	**–**	**–**	**+**	**–**
Immune deficiency	**–**	**–**	**+**	**–**	**–**	**–**	**–**
Hypoglycemia	**–**	**+**	**–/+**	**–**	**n/r**	**–**	**–**
Hyper-insulinemia	**–**	**–**	**–**	**+/–**	**n/r**	**+**	**+**
IGF-I	↓	**n/**↓	↓	**n/**↓	**n/↑**	**↓**	**↑**
IGFBP-3	↓	**n/**↓	**↓**	**n**	**n/↑**	**↓**	**↑**
ALS	**n/**↓	**n/**↓	**+/–**	**n**	**n/r**	**↓**	**↑**
GH	**↑**	**n/↑**	**↑**	**n/↑**	**n/↑**	**↑**	**↑**
GHBP deficiency	**+/–**	**–**	**–**	**–**	**–**	**–**	**–**

+, Positive; –, Negative; +/–, Predominantly positive; –/+, Predominantly negative; *, approximately 50%; n/a, not applicable; n/r, not reported; ↑, increased; ↓, decreased; n, normal; ALS, acid labile subunit; IGFBP-3, IGF binding protein-3; GHBP, growth hormone binding protein.

## GHR Mutations Associated With Mild Phenotypes

In the endocrine scientific community, there was initially some resistance to the concept of *GHR* mutations being associated with a milder phenotype than is seen in classical Laron syndrome. However, there is clear evidence that GH insensitivity of primary genetic origin is not always associated with extreme short stature (3). Evidence of a phenotypic and biochemical continuum in patients characterised as having GH insensitivity first arose from a European cohort of 82 subjects identified for replacement therapy with rhIGF-I (2). Phenotypic characteristics were strikingly broad with heights ranging from -2.2 to -10.4 SDS. An analysis of 70 subjects with features of GH insensitivity and *GHR* mutations in our laboratory showed that height SDS was significantly related to the type of *GHR* defect, with subjects having dominant negative or homozygous 6ψ pseudoexon mutations (see below) being more mildly affected than those with nonsense, missense or splice mutations (2).

### Dominant Negative GHR Mutations

Heterozygous dominant-negative *GHR* mutations were reported in seven children with growth failure and heights ranging from -2.0 to -4.2 SDS (3, 32). In addition to the short stature being milder than in Laron syndrome, none of these patients had the dysmorphic cranio-facial features of classical GH insensitivity. Facial appearances were normal or only minimally dysmorphic. All subjects had IGF-I deficiency with normal GH secretion as evidence of their GH resistance. It is understandable that such patients, if not investigated in detail, could be incorrectly categorised as having ISS and therefore potentially treated with rhGH, to which they would not be responsive (11).

### The Intronic GHR Pseudoexon Mutation

The intronic *GHR* pseudoexon mutation (6ψ) was first described in 2001 in four siblings with mild GH insensitivity from a consanguineous Pakistani family (33). The inclusion of the abnormal pseudoexon sequence in the *GHR* transcript translates to the insertion of 36 new amino acids within the extracellular domain and induces abnormal function of the mutant GHR protein. In 2018, the group from the William Harvey Research Institute in London reported 20 cases, the clear majority being from consanguineous Pakistani families (34). The mean height SDS was -4.1 ± 0.95, mean IGF-I SDS was -2.8 ± 1.4 SDS and mean IGFBP-3 SDS was -3.0 ± 2.1. Ten out of the 20 subjects (50%) had facial features consistent with classical GH insensitivity and 10 had normal facial appearance. The phenotype of 6ψ subjects is therefore variable, more so than in other extracellular *GHR* mutations and it has recently been reported that variable amounts of 6ψ- and wild type-*GHR* transcripts were identified in 6ψ patients. Higher 6ψ:wild-type *GHR* transcript ratio correlated with the severity of the short stature phenotype (35).

### IGFALS and PAPP-A2 Mutations

Additional homozygous mutations associated with relatively mild short stature, hence predisposing to incorrect categorisation as ISS, are defects of the acid-labile subunit (*IGFALS)* and pregnancy-associated plasma protein A2 (*PAPP-A2)* genes. Both these proteins have key functional roles in the transport of IGF-I and IGFBP-3 in the circulation and the regulation of IGF-I bioavailability to peripheral tissues. *IGFALS* mutations cause severe deficiency of circulating ALS, with the inability to form the ternary complex resulting in marked reduction of serum IGF-I and IGFBP-3 concentrations (2, 3). However, paradoxically, the degree of growth disturbance is mild, presumably related to generation of free IGF-I in peripheral tissues, hence preserving autocrine/paracrine IGF-I action.

In 2016, the first human cases of PAPP-A2 deficiency were described that led to increased circulating IGF-I and IGFBP-3 concentrations due to an inability of the PAPP-A2 protein to cleave the ternary complex and release free bioavailable IGF-I (29). As in ALS deficiency, the phenotype is subtle, being characterised by mild short stature (height ranging from -1.0 to -3.8 SDS), mild microcephaly, long thin nose and small chin with long fingers and toes. However, the serum concen*t*rations of (total) IGF-I, IGFBP-3 and ALS are diagnostically elevated (3).

### Heterozygous Mutations Causing Short Stature

Heterozygous mutations of key genes regulating GH action may be associated with short stature phenotypes, but these are less severe than those caused by their homozygous counterparts. Examples are mutations of the following genes; *GHR*, *STAT5B*, *IGF-I* and *IGFALS* (1, 3).

## ISS as an Indication for rhGH Therapy

When hGH was approved by the FDA in October 1985 for therapy in children with ‘inadequate GH’, this was contingent on the establishment of post-marketing surveillance. Genentech, the makers of the approved rhGH, Protropin, set up the National Cooperative Growth Study (NCGS), within which ISS was a designated diagnostic group (36). ‘Idiopathic short stature’, thus became an established label for short children with normal GH secretion, normal birth weight and absence of chromosomal defects or chronic illness and was soon adopted throughout the paediatric endocrinology community, particularly in the USA. Alternative terms were proposed such as idiopathic growth failure (IGF) or growth failure of unknown aetiology (GFUE) to change the emphasis from “stature” to “growth” or growth rate (1). However, the term ISS prevailed and thirty five years later, remains a popular designation for short children with no defined aetiology. Importantly, patients with “normal variant short stature” specifically those with familial short stature and constitutional growth delay were not excluded from the “ISS” designation.

Randomized clinical trials with rhGH were led by the Pharma Industry and produced positive growth-promoting results (37, 38) and ISS was soon referred to as ‘*a condition termed idiopathic short stature’* or a *‘diagnostic category’* (39). Positive data confirmed the effects of rhGH therapy, notably compared with placebo-treated controls (38). Predictably, these results lead to approval of ISS by the FDA in 2003 as an indication for rhGH therapy, under the criteria of height <-2.25 SD without evidence of underlying disease or GH deficiency and short expected adult height. This decision had major implications on clinical practice as suddenly 400,000 children in the USA were eligible for rhGH therapy (40). Similar applications to the European Medicines Agency were unsuccessful, due largely to the absence of data showing a positive rhGH-effect on quality of life (41).

The FDA approval for hGH therapy of ISS consolidated this category of patients in the minds of paediatricians with data on efficacy and safety accumulating in international databases such as the Kabi international growth study (KIGS) and NCGS (42). Results of cohorts of ISS subjects were, and still are, being regularly analysed and published (43, 44) and are used as the basis for management guidelines (45). ISS is also used as a diagnostic category in the ESPE and International Classifications of Pediatric Endocrine Disorders (www.icped.org) (46). Notably, the ISS patients treated with rhGH responded inconsistently and in particular, growth during year 1 of therapy did not predict the response in year 2, which emphasised the marked heterogeneity of patients carrying the ISS label (47).

## Influence of GH Resistance on Growth Response to rhGH

In clinical medicine, ‘diagnosis’ generally means that the aetiology of a condition has been identified. As stated above, ISS is not a final diagnosis and the designation ‘idiopathic’ means that no aetiology has been identified. We accept that the term ISS will continue to be used. The FDA licence for rhGH therapy in ISS increases the temptation for clinicians to use this label in order to prescribe rhGH either as short-term or long-term therapy. Doses of rhGH higher than those recommended by the FDA and EMA must not be used as this would increase the risk of adverse effects of supranormal IGF-I levels and possible non-growth related effects such as acromegaloid features (11).

However, this approach can be both psychologically damaging when an over-optimistic height prognosis is predicted to rhGH therapy and counter-productive when a pathogenesis supporting alternative therapy such as rhIGF-I would be more effective. The challenge for a clinician who is presented with a patient with short stature, who has normal GH secretion, but deserves rhGH therapy because of significant short stature, revolves around decisions of whether to treat with rhGH doses appropriate for GH deficiency or to use higher doses advised for ISS. Alternatively, should rhIGF-I be prescribed based on evidence of GH resistance?

Treatment with rhGH is usually taken as the ‘safest choice’, but may not be the best choice if the diagnosed defect is situated in a position on the continuum model GH responsiveness scale which suggests that rhGH therapy is unlikely to be beneficial (**Figure 1**). Clinicians have been hesitant to prescribe rhIGF-I as first choice in this situation. Published data on the effect of rhIGF-I in ISS subjects are extremely rare. WE would recommend that rhIGF-I is given for specific disorders where the origin and nature of the Gh resistance has been clearly demonstrated. However, if serum IGF-I is consistently low and GH secretion is normal, a diagnosis of GH resistance can be made and first-line therapy with rhIGF-I is indicated, which can be beneficial in the long-term (45).

A case in point is the molecular disorder of *PAPP-A2* mutations. Affected children have mild short stature with subtle dysmorphic features. If labelled as ISS, rhGH therapy might well be prescribed. In fact, identifying and understanding the correct pathogenesis will lead to therapy with rhIGF-I, as the mutation results in deficiency of free IGF-I, and positive responses to rhIGF-I therapy have now been reported (48, 49). The same argument applies to mild *GHR* mutations, where responses to rhIGF-I have also been documented (3, 34), in contrast to lack of evidence of responses to rhGH. Genetic identification of *IGFIR* defects can be compared to published experience of rhGH therapy in such patients (31) rather than to non-specific responses to rhGH in idiopathic SGA subjects.

## Conclusions

We have described how the term ‘idiopathic short stature’ was created, then adopted and is likely to continue to be used. Investigation of children with short stature should in our opinion follow three simultaneous lines of approach, clinical assessment, endocrine evaluation and genetic analysis. These three components should have equal status in the hierarchy of the investigational tree. This comprehensive diagnostic approach in children with short stature and normal GH secretion can give a relatively high positive diagnostic yield (50). The identification of GH resistance in a child who would otherwise be labelled as having ISS, immediately removes the patient from the ISS category and treatment can be approached on an individual basis. If rhGH is to be used for the treatment of ISS, a high dose of rhGH ~50 μg/kg/day should be used and if no growth acceleration is seen after one year, the treatment should be discontinued and alternative therapy considered (12).

## Author Contributions

All authors listed have made a substantial, direct, and intellectual contribution to the work and approved it for publication.

## Conflict of Interest

The authors declare that the research was conducted in the absence of any commercial or financial relationships that could be construed as a potential conflict of interest.

## Publisher’s Note

All claims expressed in this article are solely those of the authors and do not necessarily represent those of their affiliated organizations, or those of the publisher, the editors and the reviewers. Any product that may be evaluated in this article, or claim that may be made by its manufacturer, is not guaranteed or endorsed by the publisher.

## References

[1] Rapaport R Wit JM Savage MO. Growth Failure: ‘Idiopathic’ Only After a Detailed Diagnostic Evaluation. End Conn (2021) 10:R1–14. doi: 10.1530/EC-20-0585 PMC8052574 33543731

[2] David A Hwa V Metherell LA Netchine I Camacho-Hübner C Clark AJL. Evidence for a Continuum of Genetic, Phenotypic and Biochemical Abnormalities in Children With Growth Hormone Insensitivity. Endocr Rev (2011) 32:472–97. doi: 10.1210/er.2010-0023 21525302

[3] Storr HL Chatterjee S Metherell LA Foley C Rosenfeld RG Backeljauw PF. Nonclassical GH Insensitivity: Characterization of Mild Abnormalities of GH Action. EndocriRev (2019) 40:476–505. doi: 10.1210/er.2018-00146 PMC6607971 30265312

[4] Frasier SD. The Serum Growth-Hormone Response to Hypoglycemia in Dwarfism. J Pediatr (1967) 71:625–38. doi: 10.1016/s0022-3476(67)80197-5 6054751

[5] Goeddel DV Heyneker HL Hozumi T Arentzen R Itakura K Yansura DG. Direct Expression in Escherichia Coli of a DNA Sequence Coding for Human Growth Hormone. Nature (1979) 281:544–8. doi: 10.1038/281544a0 386136

[6] Rudman D Kutner MH Blackston RD Cushman RA Bain RP Patterson JH. Children With Normal-Variant Short Stature: Treatment With Human Growth Hormone for Six Months. N Eng J Med (1981) 305:123–31. doi: 10.1056/NEJM198107163050302 7195462

[7] Van Vliet G Styne DM Kaplan SL Grumbach MM. Growth Hormone Treatment for Short Stature. N Eng J Med (1983) 309:1016–22. doi: 10.1056/NEJM198310273091703 6684729

[8] Underwood LE. Report of the Conference on Uses and Possible Abuses of Biosynthetic Human Growth Hormone. N Eng J Med (1984) 311:606–8. doi: 10.1056/NEJM198408303110925 6379463

[9] Ranke MB. Towards a Consensus on the Definition of Idiopathic Short Stature. Horm Res (1996) 45(Suppl 2):64–6. doi: 10.1159/000184851 8805048

[10] Wit JM Clayton PE Rogol AD Savage MO Saenger PH Cohen P. Idiopathic Short Stature: Definition, Epidemiology, and Diagnostic Evaluation. GH IGF Res (2008) 18:89–110. doi: 10.1016/j.ghir.2007.11.004 18182313

[11] Wit JM Reiter EO Ross JL Saenger PH Savage MO Rogol AD. Idiopathic Short Stature: Management and Growth Hormone Treatment. GH IGF Res (2008) 18:111–35. doi: 10.1016/j.ghir.2007.11.003 18178498

[12] Cohen P Rogol AD Deal CL Saenger P Reiter EO Ross JL. Consensus Statement on the Diagnosis and Treatment of Children With Idiopathic Short Stature: A Summary of the Growth Hormone Research Society, the Lawson Wilkins Pediatric Endocrine Society, and the European Society for Paediatric Endocrinology Workshop. J Clin Endocrinol Metab (2008) 93:4210–7. doi: 10.1210/jc.2008-0509 18782877

[13] Hermanussen M Cole J. The Calculation of Target Height Reconsidered. Horm Res (2003) 59:180–3. doi: 10.1159/000069321 12649571

[14] Dauber A. Genetic Testing for the Child With Short Stature-Has the Time Come To Change Our Diagnostic Paradigm? J Clin Endocrinol Metab (2019) 104:2766–9. doi: 10.1210/jc.2019-00019 30753512

[15] Savage MO Burren CP Rosenfeld RG. The Continuum of Growth Hormone-IGF-I Axis Defects Causing Short Stature: Diagnostic and Therapeutic Challenges. Clin Endocrinol (Oxf) (2010) 72:721–8. doi: 10.1111/j.1365-2265.2009.03775 20050859

[16] Yengo L Sidorenko J Kemper KE Zheng Z Wood AR Weedon MN. GIANT Consortium Meta-Analysis of Genome-Wide Association Studies for Height and Body Mass Index in ∼700000 Individuals of European Ancestry. Hum Mol Genet (2018) 27:3641–9. doi: 10.1093/hmg/ddy271 PMC6488973 30124842

[17] Baron J Sävendahl L De Luca F Dauber A Phillip M Wit JM. Short and Tall Stature: A New Paradigm Emerges. Nat Rev Endocrinol (2015) 11:735–46. doi: 10.1038/nrendo.2015.165 PMC5002943 26437621

[18] Blum WF Alherbish A Alsagheir A El Awwa A Kaplan W Koledova E. The Growth Hormone-Insulin-Like Growth Factor-I Axis in the Diagnosis and Treatment of Growth Disorders. Endocr Conn (2018) 7:R212–22. doi: 10.1530/EC-18-0099 PMC5987361 29724795

[19] Salmon WD Daughaday WH. A Hormonally Controlled Serum Factor Which Stimulates Sulfate Incorporation by Cartilage *In Vitro* . J Lab Clin Med (1957) 49:825–36. 13429201

[20] Kaplan SA Cohen P. The Somatomedin Hypothesis 2007: 50 Years Later. J Clin Endocrinol Metab (2007) 92:4529–35. doi: 10.1210/jc.2007-0526 17986643

[21] LeRoith D. Clinical Relevance of Systemic and Local IGF-I: Lessons From Animal Models. Pediatr Endocrinol Rev (2008) 5(Suppl 2):739–43. 18317445

[22] Park P Cohen P. Insulin-Like Growth Factor (IGF-I) Measurements in Growth Hormone (GH) Therapy of Idiopathic Short Stature (ISS). GH IGF Res (2005) 15:S13–20. doi: 10.1016/j.ghir.2005,06.011 16039893

[23] Cohen P Rogol AD Howard CP Bright GM Kappelgaard AM Rosenfeld RG. American Norditropin Study Group. Insulin Growth Factor-Based Dosing of Growth Hormone Therapy in Children: A Randomized, Controlled Study. J Clin Endocrinol Metab (2007) 92:2480–6. doi: 10.1210/jc.2007-0204 17356043

[24] Buckway CK Guevara-Aguirre J Pratt KL Burren CP Rosenfeld RG. The IGF-I Generation Test Revisited: A Marker of GH Sensitivity. J Clin Endocrinol Metab (2001) 86:5176–83. doi: 10.1210/jcem.86.11.8019 11701674

[25] Selva KA Buckway CK Sexton G Pratt KL Tjoeng E Guevara-Aguirre J. Reproducibility in Patterns of IGF Generation With Special Reference to Idiopathic Short Stature. Horm Res (2003) 60:237–46. doi: 10.1159/000074038 14614229

[26] Pedicelli S Peschiaroli E Violi E Cianfarani S. Controversies in the Definition and Treatment of Idiopathic Short Stature (ISS). J Clin Res Pediatr Endocrinol (2009) 1:105–15. doi: 10.4008/jcrpe.v1i3.53 PMC3005647 21274395

[27] Sjoberg M Salazar T Espinosa C Dagnino A Avila A Eggers M. Study of GH Sensitivity in Chilean Patients With Idiopathic Short Stature. J Clin Endocrinol Metab (2001) 86:4375–81. doi: 10.1210/jcem.86.9.7850 11549678

[28] Klammt J Neumann D Gevers EF Andrew SF Schwartz ID Rockstroh D. Dominant-Negative STAT5B Mutations Cause Growth Hormone Insensitivity With Short Stature and Mild Immune Dysregulation. Nat Comm (2018) 9:2105. doi: 10.1038/s41467-018-04521-0 PMC5974024 29844444

[29] Dauber A Muñoz-Calvo MT Barrios V Domené HM Kloverpris S Serra-Juhé C. Mutations in Pregnancy-Associated Plasma Protein A2 Cause Short Stature Due to Low IGF-I Availability. EMBO Mol Med (2016) 8:363–74. doi: 10.15252/emmm.201506106 PMC4818753 26902202

[30] Işık E Haliloglu B van Doorn J Demirbilek H Scheltinga SA Losekoot M. Clinical and Biochemical Characteristics and Bone Mineral Density of Homozygous, Compound Heterozygous and Heterozygous Carriers of Three NovelMutations. Eur J Endocrinol (2017) 176:657–67. doi: 10.1530/EJE-16-0999 28249955

[31] Walenkamp MJE Robers JML Wit JM Zandwijken GRJ van Duyvenvoorde HA Oostdijk W. Phenotypic Features and Response to GH Treatment of Patients With a Molecular Defect of the IGF-I Receptor. J Clin Endocrinol Metab (2019) 104:3157–71. doi: 10.1210/jc.2018-02065 30848790

[32] Vairamani K Merjaneh L Casano-Sancho P Sanli ME David A Metherell LA. Novel Dominant-Negative GH Receptor Mutations Expands the Spectrum of GHI and IGF-I Deficiency. J Endocr Soc (2017) 1:345–58. doi: 10.1210/js.2016-1119 PMC5686656 29188236

[33] Metherell LA Akker SA Munroe PB Rose SJ Caulfield M Savage MO. Pseudoexon Activation as a Novel Mechanism for Disease Resulting in Atypical Growth-Hormone Insensitivity. Am J Hum Genet (2001) 69:641–6. doi: 10.1086/323266 PMC1235493 11468686

[34] Chatterjee S Shapiro L Rose SJ Mushtaq T Clayton PE Ten SB. Phenotypic Spectrum and Responses to Recombinant Human IGF-I (rhIGF-I) Therapy in Patients With Homozygous Intronic Pseudoexon Growth Hormone Receptor Mutations. Eur J Endocrinol (2018) 178:481–9. doi: 10.1530/EJE-18-0042 29500309

[35] Chatterjee S Cottrell E Rose SJ Mushtaq T Maharaj AV Williams J. Growth Hormone Receptor (GHR) Gene Transcript Heterogeneity may Explain Phenotypic Variability in Patients With Homozygous GHR Pseudoexon (6Ψ) Mutation. Endocr Conn (2020) 9:211–22. doi: 10.1530/EC-20-0026 PMC7077524 32061156

[36] Hintz RL. The Importance of the National Cooperative Growth Study (NCGS). In: Carel J-C, editor. Deciphering Growth. Berlin Heidelberg: Springer-Verlag (2005). p. 131–41.

[37] Hintz RL Attie KM Baptista J Roche A. Effect of Growth Hormone Treatment on Adult Height of Children With Idiopathic Short Stature. N Eng J Med (1999) 340:502–7. doi: 10.1056/NEJM199902183400702 10021470

[38] Leschek EW Rose SR Yanovski JA Troendle JF Quigley CA Chipman JJ. National Institute of Child Health and Human Development-Eli Lilly & Co. Growth Hormone Collaborative Group. J Clin Endocrinol Metab (2004) 89:3140–8. doi: 10.1210/jc.2003-031457 15240584

[39] Cohen P. Controversy in Clinical Endocrinology: Problems With Reclassification of Insulin-Like Growth Factor I Production and Action Disorders. J Clin Endocrinol Metab (2006) 91:4235–6. doi: 10.1210/jc.2006-1641 16954153

[40] Swatz Topor L Feldman HA Bauchner H Cohen L. Variation in Methods of Predicting Adult Height for Children With Idiopathic Short Stature. Pediatrics (2010) 126:937–44. doi: 10.1542/peds.2009-3649 PMC3793344 20974789

[41] Ranke MB Wit JM. Growth Hormone - Past, Present and Future. Nat Rev Endocrinol (2018) 14:285–300. doi: 10.1038/nrendo.2018.22 29546874

[42] Ranke MB Lindberg A Price DA Darendeliler F Albertsson-Wikland K Wilton P. KIGS International Board. Age at Growth Hormone Therapy Start and First-Year Responsiveness to Growth Hormone Are Major Determinants of Height Outcome in Idiopathic Short Stature. Horm Res (2007) 68:53–62. doi: 10.1159/000098707 17228181

[43] Kaplowitz PB Shulman DI Frane JW Jacobs J Lippe B. Characteristics of Children With Best and Poorest First- and Second-Year Growth During rhGH Therapy: Data From 25years of the Genentech National Cooperative Growth Study (NCGS). Int J Pediatr Endocrinol (2013) 9:2013–9. doi: 10.1186/1687-9856-2013-9 PMC3660178 23631505

[44] Sävendahl L Polak M Backeljauw P Blair J Miller BS Rohrer TR. Treatment of Children With GH in the United States and Europe: Long-Term Follow-Up From NordiNet® IOS and ANSWER Program. J Clin Endocrinol Metab (2019) 104:4730–42. doi: 10.1210/jc.2019-00775 PMC6812718 31305924

[45] Grimberg A Allen DB. Growth Hormone Treatment for Growth Hormone Deficiency and Idiopathic Short Stature: New Guidelines Shaped by the Presence and Absence of Evidence. Curr Opin Pediatr (2017) 29:466–71. doi: 10.1097/MOP.0000000000000505 PMC5565215 28525404

[46] International Classification of Pediatric Endocrine Diagnoses (2016). Available at: www.icped.org. 10.1159/000448893 PMC5296918 27591798

[47] Deodati A Cianfarani S. Impact of Growth Hormone Therapy on Adult Height of Children With Idiopathic Short Stature: Systematic Review. Br Med J (2011) 342:c7157. doi: 10.1136/bmj.c7157 21398350

[48] Cabrera-Salcedo C Mizuno T Tyzinski L Andrew M Vinks AA Frystyk J. Pharmacokinetics of IGF-I in PAPP-A2-Deficient Patients, Growth Response, and Effects on Glucose and Bone Density. J Clin Endocrinol Metab (2017) 102:4568–77. doi: 10.1210/jc.2017-01411 PMC5718699 29029190

[49] Muñoz-Calvo MT Barrios V Pozo J Chowen JA Martos-Moreno GÁ Hawkins F. Treatment With Recombinant Human Insulin-Like Growth Factor-1 Improves Growth in Patients With PAPP-A2 Deficiency. J Clin Endocrinol Metab (2016) 101:3879–83. doi: 10.1210/jc.2016-2751 PMC5393598 27648969

[50] Andrews A Maharaj A Cottrell E Chatterjee S Shah P Denvir L. Genetic Characterization of Short Stature Patients Presenting With Phenotypic and Endocrine Overlap of Known Growth Hormone Insensitivity Syndromes. J Clin Endocrinol Metab (2021) 106:e4716–33. doi: 10.1210/clinem/dgab437 PMC8530715 34136918

